# Eligibility criteria for clinical trials in AL amyloidosis result in exclusion of nearly half of real‐world patients

**DOI:** 10.1002/hem3.70320

**Published:** 2026-02-16

**Authors:** Claudia Bellofiore, Marco Basset, Mario Nuvolone, Martina Nanci, Giuseppe D. Sanna, Valeria A. Di Simone, Alessandro Fogliani, Monica Di Cecca, Roberta Mussinelli, Andrea Foli, Matin Ghazi Esfahani, Mohammadjavad Sadrzadeh, Stefano Perlini, Leonardo De Luca, Solène Clavreul, Giampaolo Merlini, Giovanni Palladini, Paolo Milani

**Affiliations:** ^1^ Department of Molecular Medicine University of Pavia Pavia Italy; ^2^ Department of Hematology A.O.U. Policlinico Gaspare Rodolico of Catania Catania Italy; ^3^ Amyloidosis Research and Treatment Center, Fondazione IRCCS Policlinico San Matteo Pavia Italy; ^4^ Clinical and Interventional Cardiology Sassari University Hospital Sassari Italy; ^5^ Division of Cardiology Fondazione IRCCS Policlinico San Matteo Pavia Italy; ^6^ Hematology, Stem Cell Transplantation Fondazione Policlinico Universitario Campus Bio medico di Roma Rome Italy; ^7^ Department of Internal Medicine University of Pavia Pavia Italy; ^8^ Myeloma Patients Europe Brussels Belgium

Immunoglobulin light chain (AL) amyloidosis is a rare and potentially life‐threatening disorder characterized by the deposition of misfolded, toxic immunoglobulin light chains in organs and tissues, typically produced by a plasma cell (PC) clone.^1^ The deposition of amyloidogenic light chains disrupts tissue structure, leading to organ dysfunction with a wide range of symptoms and clinical manifestations.^1^, ^2^, ^3^ Any organ can be involved, except the central nervous system, with the heart being the key prognostic factor for survival.^2^ Early diagnosis and treatment initiation are crucial to halt organ damage and improve patients' outcomes.^2^ Currently, the treatment strategy relies on drugs directed against the underlying PC clone to reduce the production of amyloidogenic light chains.^2^

To date, only a few clinical trials of upfront anti‐PC therapies have been completed.^4^, ^5^, ^6^, ^7^, ^8^ Until the results of the phase III clinical studies comparing the combination therapy bortezomib, melphalan, and dexamethasone (BMDex) versus melphalan and dexamethasone (MDex)(NCT01277016)^4^ and the ANDROMEDA trial (NCT03201965), which led to the approval of the current standard of care,^5^ clinical evidence for the treatment of AL amyloidosis was primarily based on retrospective studies.^9^, ^10^

In this heterogeneous disease, clinical trial inclusion criteria can select subgroups of patients that differ from the real‐world population, affecting the generalizability of results. Eligibility criteria aim to create homogeneous study populations to enhance internal validity and minimize confounding factors; however, they may exclude certain groups or clinical presentations that are common in the real‐world setting.^11^, ^12^ As recently reported, most of the clinical trials for AL amyloidosis excluded patients with severely compromised hearts (IIIb cardiac stage).^8^ Only lately, a phase III clinical trial evaluating the effects of anti‐fibrillary agents on overall survival (OS) included cardiac stage IIIb patients (NCT04504825).

In this study, we aimed to explore the proportion of newly diagnosed patients with AL amyloidosis who are ineligible for phase III trials and to investigate how common exclusion criteria affect outcomes in the real‐world population.

The prospectively maintained ReAL registry (NCT04839003), which enrolls all patients with a confirmed diagnosis of AL amyloidosis evaluated at the Pavia Amyloidosis Center, was searched for newly diagnosed patients with AL amyloidosis treated from 2004 to 2021. All patients gave written informed consent before inclusion in ReAL. Patients with myeloma‐defining events, per International Myeloma Working Group criteria, were excluded.^13^ The clinicaltrials.gov platform was searched for frontline phase III clinical trials for AL amyloidosis initiated between 2002 and 2021. The following clinical trials were identified: NCT01078454, NCT01277016, NCT03201965. The eligibility criteria from these trials were extracted, and only the criteria that were common across all three studies were selected. Patients included in the study were divided into two cohorts according to their eligibility for the considered phase III clinical trial. Patients with at least one exclusion criterion were considered not eligible. Patients were further divided based on whether they initiated first‐line treatment before or after 2010. This division was made to capture changes in disease management following the introduction of bortezomib‐based regimens in the treatment landscape after 2010. Among patients deemed ineligible due to advanced heart involvement (IIIb cardiac stage), a subanalysis was conducted, dividing them into two groups based on whether they met at least one exclusion criterion for the phase III clinical trial NCT04504825 evaluating the use of antifibrillary agents. Hematologic response was evaluated according to the International Society of Amyloidosis criteria.^14^

All variables were tested for normality by Shapiro−Wilk's test. Fisher's exact test and Mann−Whitney test were used to assess differences in nominal and continuous variables between groups, as appropriate. Results were expressed as median and interquartile range (IQR) for continuous variables, or as number of cases and percentage for categorical variables. Overall survival was calculated from diagnosis to death or last contact. Survival curves were plotted according to Kaplan−Meier, and differences in OS were tested for significance with the log‐rank test. Statistical analysis was conducted with MedCalc® Statistical Software version 20.027 (MedCalc Software Ltd., Ostend, Belgium; https://www.medcalc.org; 2022).

A cohort of 1726 consecutive patients was considered for the analysis (Table 1). Among them, 734 (42%) subjects were found to meet at least one exclusion criterion of the considered phase III clinical trials. The main reasons for their ineligibility to clinical trials were IIIb cardiac stage (44%), Eastern Cooperative Oncology Group performance status (PS‐ECOG) > 2 (35%), and estimated glomerular filtration rate (eGFR) <30 mL/min/1.73 m^2^ (23%). Among patients older than 75 years of age, the most common causes of ineligibility were consistent: IIIb cardiac stage (54%), PS‐ECOG > 2 (40%), and eGFR <30 mL/min/1.73 m^2^ (34%). Bortezomib‐based regimens were more commonly used as first‐line treatments in patients eligible for clinical trials (60% vs. 49%, *P* < 0.001). Overall, a higher rate of hematologic response was observed in patients eligible for clinical trials [overall response rate (ORR) 45% vs. 31%, *P* < 0.001] with a higher proportion of patients achieving a very good partial response (VGPR) or better (36% vs. 22%, *P* < 0.001). When limiting the analysis of hematologic response rates to patients receiving upfront bortezomib‐based regimens, the significant advantage for eligible patients persisted (49% vs 31%, *P* < 0.001; ≥VGPR 39% vs. 23%, *P* < 0.001).

**Table 1:** Patients' baseline characteristics.

	Not eligible (*N* = 734) Median (IQR) *N* (%)	Eligible (*N* = 992) Median (IQR) *N* (%)	*P*
Age, years	67 (59–73)	65 (56–72)	0.003
Male sex	427 (58)	574 (57)	0.896
BMPC, %	12 (7–20)	10 (7–17.5)	0.043
dFLC mg/L	236 (107–547)	134 (53–366)	<0.001
λ:κ	545 (74): 189 (26)	795 (80): 197 (20)	0.003
NT‐proBNP ng/L	9994 (9183–10 815)	1321 (1121–1554)	<0.001
cTnI ug/L	0.137 (0.043–0.300)	0.038 (0.035–0.042)	<0.001
Proteinuria g/24 h	1.0 (0.24–5.1)	2.5 (0.3–6.5)	0.003
eGFR mL/min/1.73 m^2^	51 (25–>60)	75 (53–>60)	<0.001
Abnormal ALP	196 (26)	151 (15)	<0.001
Organ involvement			
Heart	658 (89)	659 (66)	<0.001
Kidney	439 (60)	679 (68)	<0.001
Liver	112 (15)	96 (10)	<0.001
>2 organs	218 (30)	122 (12)	<0.001
Cardiac stage^a^			
I/II/IIIa/IIIb	40 (5)/170 (23)/170 (23)/322 (44)	234 (23)/448 (45)/249 (25)/0 (0)	<0.001
Renal stage^b^			
I/II/III	296 (40)/295 (40)/107 (14)	517 (52)/380 (38)/95 (9)	<0.001
Dialysis at diagnosis	36 (5)	0 (0)	
Patients diagnosed after 2010	508 (69)	767 (77)	<0.001
Common clinical trial exclusion criteria			
eGFR< 30 mL/min/1.73 m^2^	172 (23)		
Orthostatic hypotension	130 (18)		
NYHA ≥ IV	27 (4)		
PS‐ECOG > 2	257 (35)		
IIIb cardiac stage	322 (44)		

Abbreviations: ALP, alkaline phosphatase; ANS, autonomic nervous system; BMPC, bone marrow plasma cell; cTnI, cardiac troponin‐I; dFLC, difference between involved minus uninvolved serum‐free light chains; eGFR, estimated glomerular filtration rate; IQR, interquartile range; NT‐proBNP, N‐terminal pro‐B‐type natriuretic peptide; PNS, peripheral nervous system.

^a^ Cardiac stage based on troponin and NT‐proBNP levels: thresholds for cTnI (or hs‐cTnI) and NT‐proBNP are <0.1 μg/L (<77 ng/L) and <332 ng/L, respectively. Stage IIIa cardiac involvement is defined by cTnI > 0.1 ug/L (or hs‐cTnI > 77 ng/L) and NT‐proBNP > 332 ng/L, provided that NT‐proBNP < 8500 ng/L. Stage IIIb cardiac involvement is defined by cTnI > 0.1 ug/L (or hs‐cTnI > 77 ng/L) and NT‐proBNP ≥ 8500 ng/L. Stage II patients have one value of either troponin or NT‐proBNP above the thresholds. Stage I patients have troponin and NT‐proBNP below the thresholds.

^b^ Renal stage based on proteinuria and estimated glomerular filtration rate (eGFR) levels: thresholds for proteinuria > 5 g/24 h and eGFR < 50 mL/min/1.73 mq; stage I, both proteinuria ≤ 5 g/24 h and eGFR ≥ 50 mL/min/1.73 mq; stage II, either proteinuria > 5 g/24 h or eGFR < 50 mL/min/1.73 mq; stage III, both proteinuria > 5 g/24 h and eGFR < 50 mL/min/1.73 mq.

The median follow‐up of the living patients in the entire cohort was 106 months (95% confidence interval [95% CI] 95–234 months). Eligible patients demonstrated a substantially longer median OS compared to their ineligible counterparts (68 vs. 9 months, *P* < 0.001), as reported in Figure 1. Regardless of the cardiac stage, eligible patients consistently exhibited prolonged median OS compared to ineligible patients (Figure 1). In the eligible cohort, significantly lower mortality rates at 24 months from diagnosis were observed across all cardiac stages: stage I (11% vs. 20%, *P* = 0.022), stage II (30% vs. 37%, *P* = 0.019), and stage IIIa (47% vs. 72%, *P* < 0.001). Moreover, subgroup analysis focusing on patients enrolled after 2010 revealed similar patterns of survival benefit across different cardiac stages. In this subgroup, eligible patients consistently showed significantly lower 24‐month mortality rates compared to ineligible patients: stage I (7% vs. 28%, *P* = 0.023), stage II (30% vs. 40%, *P* = 0.039), and stage IIIa (52% vs. 74%, *P* < 0.001) (Figure 1).

**Figure 1:**
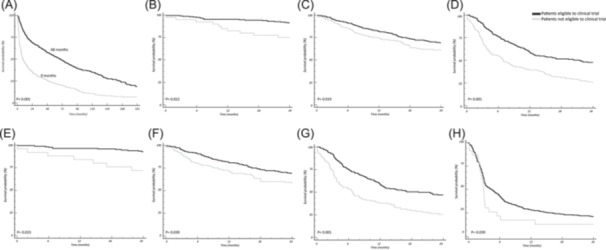
**(A) Median overall survival (OS) according to eligibility to phase III clinical trial; (B) median OS in patients with Mayo Clinic/European cardiac stage I; (C) median OS in patients with Mayo Clinic/European cardiac stage II; (D) median OS in patients with Mayo Clinic/European cardiac stage IIIa; (E) median OS in patients with Mayo Clinic/European cardiac stage I treated after 2010; (F) median OS in patients with Mayo Clinic/European cardiac stage II treated after 2010; (G) median OS in patients with Mayo Clinic/European cardiac stage IIIa treated after 2010; (H) median OS in patients with Mayo Clinic/European cardiac stage IIIb according to eligibility to phase III clinical trial**.

Finally, the study population comprised 322 patients (18%) with IIIb cardiac stage. Among these, 23 patients (7%) were classified as ineligible for the phase III clinical trial NCT04504825 due to the presence of severe hypotension (supine systolic blood pressure <90 mmHg) and/or symptomatic orthostatic hypotension. No significant differences in age were observed between the two cohorts (Supporting Information S1: Table 1B). Ineligible patients demonstrated significantly lower OS compared to eligible ones (2.9 vs. 4.5 months, *P* = 0.039).

To the best of our knowledge, this study is the first to examine the impact of front‐line AL amyloidosis clinical trial eligibility criteria on patient demographics, the proportion of excluded subjects, and patient outcomes. We showed that the eligibility criteria adopted in current phase III clinical trials exclude 40% of real‐world subjects. The primary reasons for exclusion were advanced cardiac involvement (stage IIIb), poor performance status (PS‐ECOG > 2), and renal impairment (eGFR<30 mL/min/m^2^). Eligible patients showed higher hematologic response rates than ineligible ones, yet response rates were lower than those reported in randomized trials,^4^, ^5^ likely reflecting the less selected nature of this real‐world cohort. Similarly, in multiple myeloma, up to 45% of newly diagnosed patients are trial‐ineligible, which is consistently associated with inferior survival.^15^ Clinical trial eligibility criteria, aimed at protecting patient safety and ensuring internal validity, may compromise the generalizability of trial findings by excluding a broad spectrum of patients. On the one hand, this limitation poses significant challenges for regulatory agencies, which must base reimbursement decisions on evidence that may not reflect the real‐world population, potentially limiting access to new therapies for a substantial proportion of patients. Yet, on the other hand, extending the use of new treatments to a broader, unselected population carries the risk of exposing the more fragile patients to higher toxicity and achieving worse outcomes. However, if tolerability and dosing strategies are not assessed in fragile populations during trials, standardizing safe treatment approaches after approval becomes more complex. This underscores the need for trial designs that incorporate dose‐escalation strategies or dedicated subgroups to better inform clinical decision‐making and improve treatment access without compromising patient safety. In this context, frailty assessment could be valuable, as recently reported.^16^ Integrating frailty scores into trial designs may enhance patients' stratification by identifying those with a higher risk of treatment‐related toxicity. Besides, dedicated trials for patients with advanced cardiac involvement are also crucial to optimize therapy for this high‐risk population. Additionally, real‐world evidence studies and observational cohorts should be leveraged to complement clinical trial data and provide insights into the effectiveness and safety of treatments in a diverse patient population. Retrospective studies have already driven critical advancements in the field, including improved staging systems and response criteria, which have significantly shaped the field of AL amyloidosis.^14^, ^17^, ^18^, ^19^, ^20^ In this context, prospectively maintained registries such as national single‐center registries (e.g., NCT4839003) and international registries (e.g., NCT06383143 and NCT06205953) can offer valuable resources to achieve this aim. In conclusion, our study highlights the critical impact of clinical trial eligibility criteria on patient selection, treatment responses, and survival in AL amyloidosis. By broadening eligibility criteria and incorporating real‐world evidence, future research can provide more comprehensive and applicable insights into the optimal management of this disease.

## AUTHOR CONTRIBUTIONS

**Claudia Bellofiore:** Writing—original draft; investigation; data curation; formal analysis. **Marco Basset:** Investigation. **Mario Nuvolone:** Investigation. **Martina Nanci:** Investigation. **Giuseppe D. Sanna:** Data curation. **Valeria A. Di Simone:** Data curation. **Alessandro Fogliani:** Investigation. **Monica Di Cecca:** Investigation. **Roberta Mussinelli:** Investigation. **Andrea Foli:** Investigation. **Matin Ghazi Esfahani:** Data curation. **Mohammadjavad Sadrzadeh:** Data curation. **Stefano Perlini:** Data curation. **Leonardo De Luca:** Data curation. **Solène Clavreul:** Writing—review and editing. **Giampaolo Merlin:** Investigation; writing—original draft; writing—review and editing; supervision. **Giovanni Palladini:** Investigation; methodology; writing—original draft; supervision. **Paolo Milani:** Investigation; methodology; writing—original draft.

## CONFLICT OF INTEREST STATEMENT

G.P. received honoraria from Pfizer, Sebia, and Siemens and served on advisory boards for Alexion, Argobio, GSK, Janssen, and Prothena. P.M. has received honoraria from Janssen, Prothena, Pfizer (also received a research grant), and Siemens (also served on an advisory board). M.N. has received honoraria from Janssen, Prothena, Pfizer (also research grant), and Argobio, and has served on the advisory board and received a research grant from Gate Bioscience.

## FUNDING

This work was supported by grants from the European Union—Next Generation EU—PNRR M6C2—Investment 2.1 “Valorizzazione e potenziamento della ricerca biomedica del SSN” (grant #PNRR‐MR1‐2022‐12376853), the Italian Ministry of Health (Ricerca Finalizzata, grant #GR‐2018‐12368387, and Ricerca Corrente), the Italian Ministry of Research and Education (PRIN 20207XLJB2), the CARIPLO Foundation (grant #2018‐0257), the CARIPLO Foundation and Telethon Foundation (grant #GJC23044), Cancer Research UK 4013 (C355/A26819), FC AECC and AIRC under the Accelerator Award Program. Open access publishing facilitated by Universita di Pavia, as part of the Wiley – CRUI‐CARE agreement.

## Supporting information

Supplementary.

## Data Availability

For original data, please contact giovanni.palladini@unipv.it. The study was presented as an abstract at the XIX International Symposium on Amyloidosis (ISA) 2024.
