# Supplementary material for: Eligibility criteria for clinical trials in AL amyloidosis result in exclusion of nearly half of real‐world patients
Source: Hemasphere. 2026 Feb 16;10(2):e70320. doi: 10.1002/hem3.70320 (PMC12908192; doi:10.1002/hem3.70320)
Supplement: Supplementary file 1 — Supplementary. [file HEM3-10-e70320-s001.docx]

**Supplementary**

**Table 1.** Baseline characteristics of patients in IIIa (A) and IIIb (B) Mayo Clinic/European cardiac stage.

**A**
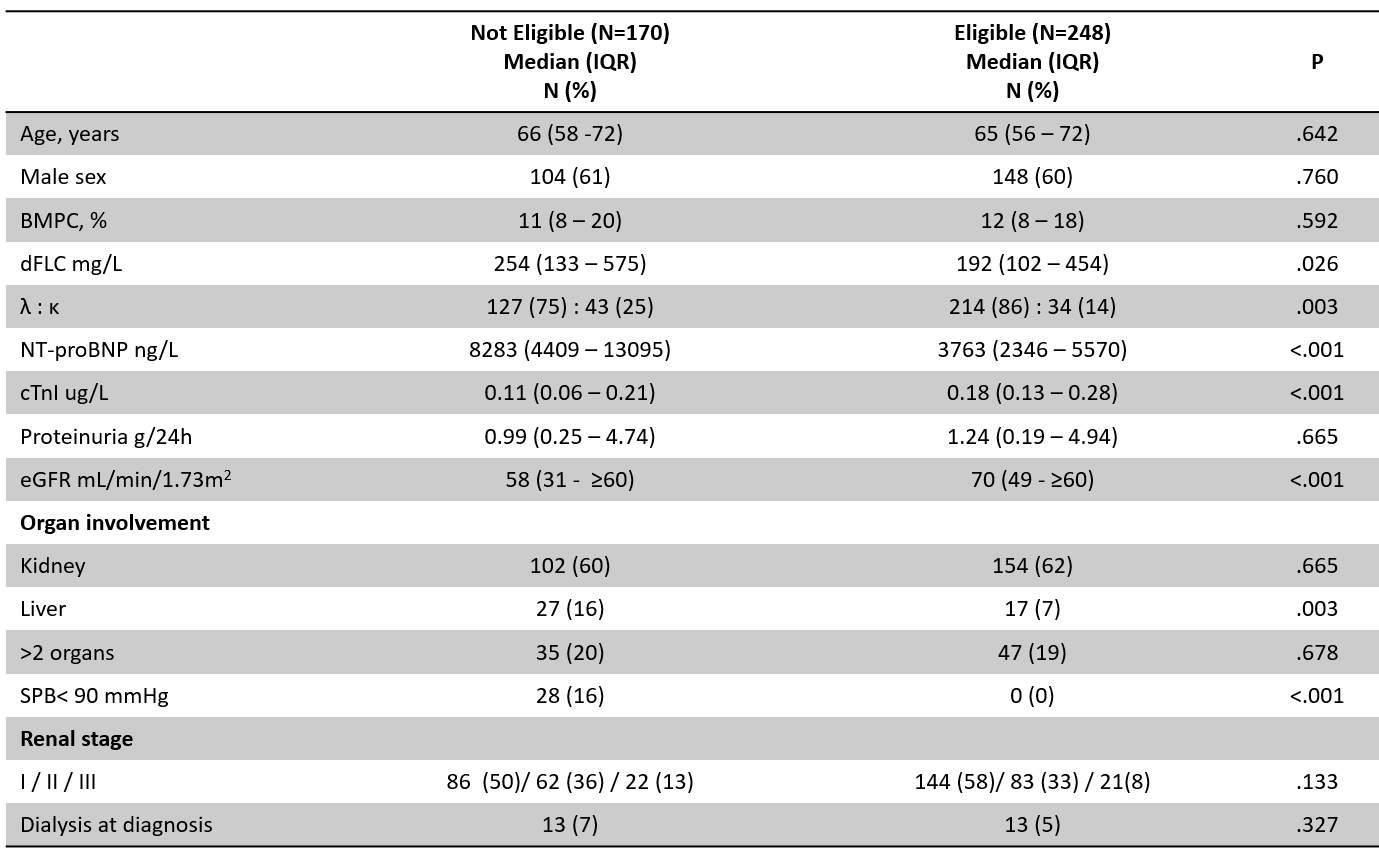


**B
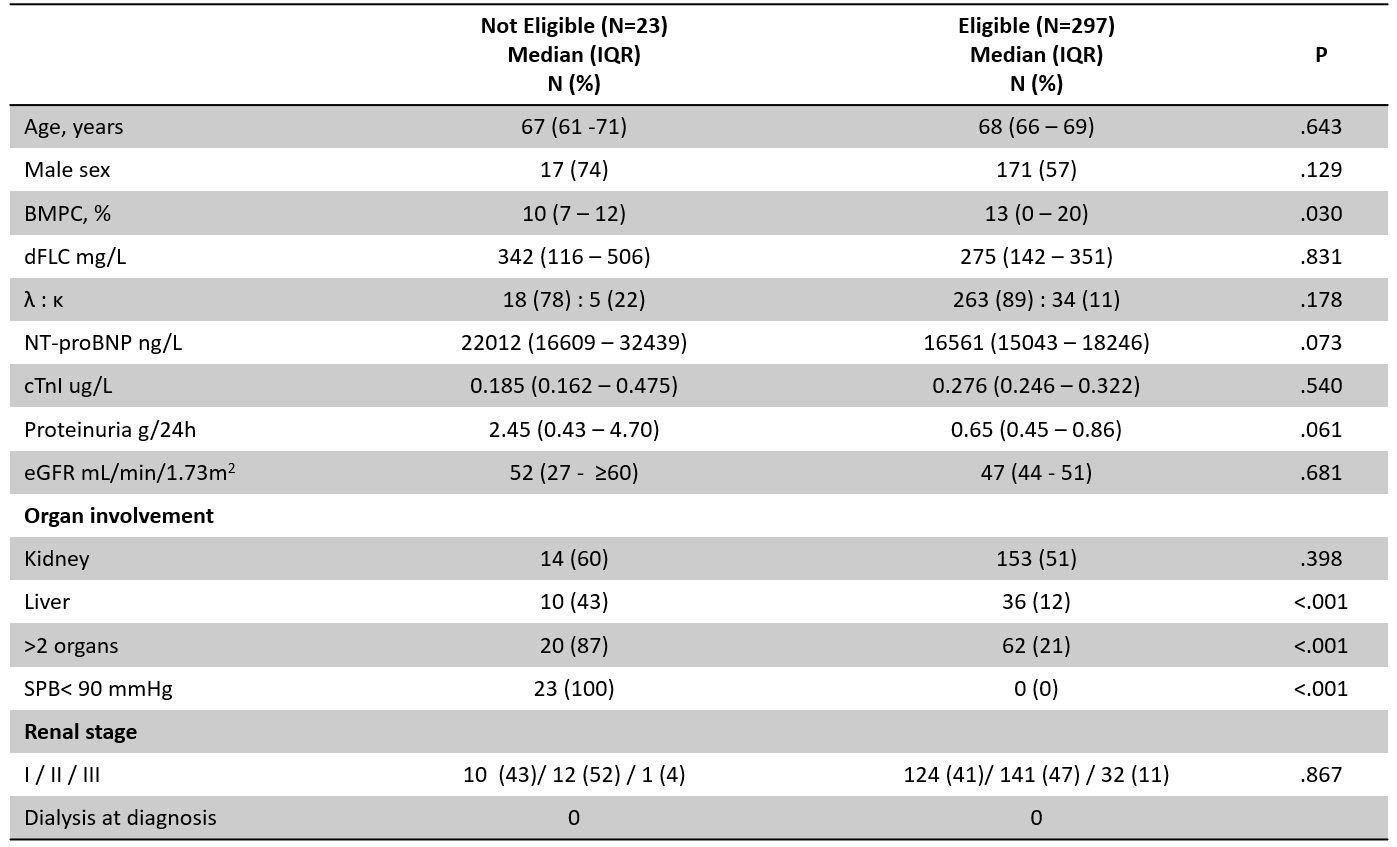
**

Legend: ANS, autonomic nervous system; BMPC, bone marrow plasma cell; cTnI, cardiac troponin-I; dFLC, difference between involved minus uninvolved serum free light chains; eGFR, estimated glomerular filtration rate; NT-proBNP, N-terminal pro-B-type natriuretic peptide; SBP, systolic blood pressure.

*Renal stage based on proteinuria and estimated glomerular filtration rate (eGFR) levels: thresholds for proteinuria >5g/24h and eGFR<50 mL/min/1.73mq Stage I, both proteinuria ≤5g/24h and eGFR≥50 mL/min/1.73mq; stage II, either proteinuria >5g/24h or eGFR<50 mL/min/1.73mq; stage III, both proteinuria >5g/24h and eGFR< 50mL/min/1.73mq.
